# Analyzing the ‘bradykinesia complex’ in GBA1-associated Parkinson's disease: A series of three cases

**DOI:** 10.1016/j.prdoa.2026.100462

**Published:** 2026-06-11

**Authors:** Martina De Riggi, Daniele Birreci, Adriana Martini, Simone Aloisio, Luca Angelini, Anna Sofia Grandolfo, Matteo Bologna

**Affiliations:** a Department of Human Neurosciences, Sapienza University of Rome, Rome, Italy; b IRCCS Neuromed, Pozzilli, (IS), Italy; c Department of Neurology and Stroke Unit, San Camillo de Lellis, General District Hospital, Rieti, Italy

**Keywords:** Parkinson's disease, GBA1, Kinematic analysis, Finger tapping, Bradykinesia complex, movement disorders, Genotype–phenotype correlation

## Abstract

“Although GBA1-associated Parkinson's disease shows distinctive clinical features, the characterization of motor impairment remains limited. We performed OFF- and ON-state kinematic finger-tapping analysis in three Parkinson's disease patients carrying GBA1 variants of different severity, applying the ‘bradykinesia complex’ framework. Quantitative assessment revealed heterogeneous kinematic profiles and variable dopaminergic responsiveness. These findings suggest that kinematic biomarkers may complement clinical evaluation and support phenotypic characterization in GBA1-associated Parkinson's disease.”

Heterogeneous variants in the glucocerebrosidase gene (GBA1) represent the most common genetic risk factor for Parkinson's disease (PD) and are associated with distinctive clinical features [1], [2], [3]. Although GBA1 carriers show earlier disease onset and increased cognitive and neuropsychiatric burden [1], [2], [3], detailed quantitative characterization of the motor phenotype remains limited. Kinematic analysis enables objective assessment of motor performance beyond standard bedside evaluation. Recent studies have shown that bradykinesia represents a complex of motor abnormalities, the ‘bradykinesia complex’, comprising partially independent motor alterations with distinct pathophysiological substrates and variable responsiveness to therapy [4]. Based on this framework, our group previously established normative cut-off values and characterized the typical kinematic profile of idiopathic PD [4]. While shared dopaminergic involvement might suggest a broadly homogeneous motor impairment across GBA1 carriers, variant-specific biological effects on lysosomal dysfunction and neurodegenerative burden could translate into distinct kinematic profiles and differential responsiveness to levodopa. Characterizing kinematic motor features may help refine the phenotypic and diagnostic characterization of GBA1-associated PD compared with idiopathic PD, while also capturing variability among GBA1 carriers that could support future prognostic stratification and therapeutic tailoring. In this exploratory case series, we report OFF- and ON-state kinematic assessments in three PD patients carrying GBA1 variants of different severity. Our aim was to provide additional phenotypic characterization and generate hypotheses on motor heterogeneity in GBA1-associated PD. Molecular genetic testing was performed in all three patients because of early-onset PD (Supplementary Materials). Variant severity was assigned according to the recent systematic review by Rossi et al. [2], which classifies GBA1 variants on the basis of available genotype–phenotype evidence. Finger-tapping (FT) performance was assessed by kinematic analysis performed according to standardized protocols, focusing on the most affected side as defined by MDS-UPDRS part III scores (Supplementary Materials) [4].

Case 1 was a 46-year-old right-handed man carrying a severe heterozygous GBA1 p.Leu444Pro (L444P) variant [2], with a positive family history of GBA-associated PD (brother). Clinical and demographic characteristics are reported in Table 1. Despite preserved global cognition and good clinical response to dopaminergic therapy, kinematic analysis in the OFF state revealed decreased velocity (bradykinesia), reduced movement amplitude (hypokinesia) and impaired rhythm regularity (dysrhythmia), with no improvement after dopaminergic medication (Fig. 1, Table 2).

**Table 1:** Clinical characteristics of GBA1-associated Parkinson's Disease (PD) patients in OFF and ON medication States.

	**Case 1**	**Case 2**	**Case 3**
Age (Onset)	46(40)	51(47)	54(52)
Sex	M	F	F
Education (y)	13	18	18
Other CNS-active medications	None	None	None
Onset symptoms	Left-sided bradykinesia	Right-sided bradykinesia and resting tremor	Left-sided resting tremor
Disease duration	6	4	2
H&Y Scale	2	2	2
LEDD	610	570	300
GBA1 variant	c.1448 T > C (p.Leu444Pro, L444P)	c.1226 A > G (p.Asn409Ser, N409S)	c.887G > A (p.Arg296Gln, R296Q)
Brain MRI	Unremarkable	Frontal–temporal and hippocampal atrophy	Unremarkable
HAM-A	2(1)	13(12)	20(20)
HAM-D	1(1)	6(5)	12(12)
MOCA*	29(29)	24(24)	30(30)
FAB	18(18)	17(17)	18(18)
UPDRS Part III	23(8)	25(13)	21(17)

**Clinical and demographic features of three patients with GBA1-associated PD evaluated in OFF and ON dopaminergic medication states.** Age, sex, age at onset, disease duration, levodopa equivalent daily dose (LEDD), and GBA1 variants are reported. Anxiety and depressive symptoms were assessed using the Hamilton Anxiety Rating Scale (HAM-A) and Hamilton Depression Rating Scale (HAM—D). Cognitive function was evaluated with the Montreal Cognitive Assessment (MoCA) and Frontal Assessment Battery (FAB). Motor severity was assessed using the Unified Parkinson's Disease Rating Scale Part III (UPDRS-III). Age is reported as age at evaluation (age at disease onset). All scale scores are presented as OFF medication (outside parentheses) and ON medication (within parentheses). *Detailed MoCA subscores are provided in the Supplementary Material. CNS, central nervous system; y, years; H&Y scale, The Modified Hoehn and Yahr Scale.

**Fig. 1:**
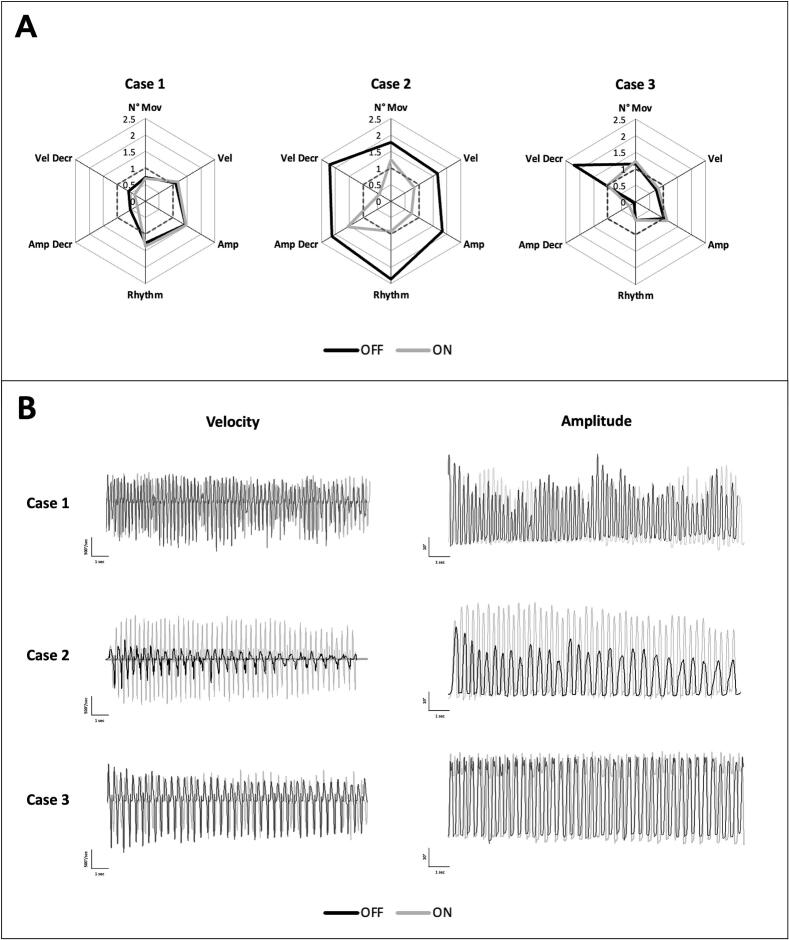
**Radar plots and kinematic traces of finger tapping (FT) parameters in GBA1-associated Parkinson's disease (PD) patients.** **Panel A.** Radar plots illustrate FT kinematic profiles in OFF and ON dopaminergic medication states for each case. All parameters were normalized to their respective pathological cut-off values to obtain unidirectional severity scores. For variables in which higher values indicate abnormality, values were divided by their respective cut-off, whereas for variables in which lower values indicate abnormality, the cut-off was divided by the observed value. A normalized score of 1 corresponds to the pathological threshold, values >1 indicate pathological performance, and values <1 indicate preserved performance. The dashed circle represents the cut-off threshold. N° Mov, number of movements; Vel, velocity; Amp, amplitude; Vel Decr, velocity decrement; Amp Decr, amplitude decrement. **Panel B.** Kinematic traces of movement velocity (left) and amplitude (right) in the three cases (Cases 1–3), recorded in OFF (black) and ON (gray) conditions. The traces illustrate both inter-individual variability and intra-individual changes between conditions, highlighting condition-dependent modulation of motor performance.

**Table 2:** Kinematic parameters of finger tapping (FT) in GBA1-Associated Parkinson's Disease (PD) patients in OFF and ON medication states.

	**Cut-off values**	**Case 1**		**Case 2**		**Case 3**	
		OFF	ON	OFF	ON	OFF	ON
Number of Mov	< 46.17	64.67	67.67	**26**	**36.67**	**40.33**	**37.67**
Rhythm (CV)	> 0.11	**0.14**	**0.15**	**0.26**	0.10	0.06	0.06
Amplitude	< 46.69	**32.35**	**31.95**	**25.55**	62.71	**46.43**	**41.22**
Velocity	< 952.33	**869.74**	**804.79**	**569.79**	1143.79	1279.83	1163.10
Amplitude Decr	> −0.17	−0.09	−0.05	**−0.36**	**−0.27**	0.01	−0.04
Velocity Decr	> −6.92	−4.05	−2.73	**−15.29**	−2.92	**−15.29**	**−6.93**

Parameters include number of movements, rhythm variability (coefficient of variation, CV), movement amplitude, movement velocity, amplitude decrement, and velocity decrement. Previously established pathological cut-off values are reported for each parameter. Changes from OFF to ON were assessed descriptively. Improvement was defined a priori as a shift toward the non-pathological direction relative to the previously established cut-off. For parameters considered abnormal when reduced (number of movements, amplitude, and velocity), improvement corresponded to higher ON values than OFF values. For parameters considered abnormal when increased (rhythm variability, amplitude decrement, and velocity decrement), improvement corresponded to a shift toward the normal range, i.e., lower rhythm variability and decrement values closer to zero. Threshold crossing was considered normalization, whereas favorable directional changes not crossing the threshold were considered partial improvement. Values falling within the pathological range, either below or above the relevant pathological threshold depending on the parameter, are highlighted in bold. Movement velocity is expressed in degrees/s and movement amplitude in degrees. Velocity and amplitude slopes are expressed in (degrees/s)/n° mov and degrees/n° mov, respectively. Number of Mov, number of movements; Vel Decr, velocity decrement; Amp Decr, amplitude decrement.

Case 2 was a 51-year-old right-handed woman carrying a mild heterozygous GBA1 p.Asn409Ser (N409S) variant [2], with no family history of neurodegenerative disorders. Clinical and demographic characteristics are reported in Table 1, where mild cognitive impairment and relevant affective symptoms were noted. In the OFF state, kinematic analysis showed marked abnormalities across all examined parameters, indicating severe impairment of movement performance (Fig. 1, Table 2). In the ON state, a substantial improvement was observed, reflecting robust dopaminergic responsiveness. This profile is consistent with the preserved dopaminergic sensitivity typically reported in carriers of mild GBA1 variants, despite relevant non-motor symptom burden.

Case 3 was a 54-year-old right-handed woman carrying a pathogenic heterozygous GBA1 p.Arg296Gln (R296Q) variant, associated with an intermediate-to-severe phenotype [2], and reporting a positive family history of dementia with Lewy bodies (mother), PD (paternal grandmother), and Alzheimer's disease (maternal grandmother). Clinical and demographic characteristics are reported in Table 1, where preserved global cognition in the presence of relevant affective symptoms was noted. Kinematic analysis in the OFF state revealed decreased amplitude (hypokinesia), and marked progressive velocity decrement (sequence effect), which showed minimal change in the ON state (Fig. 1, Table 2), reflecting limited dopaminergic responsiveness. Overall, this profile suggests early motor involvement despite short disease duration.

In this exploratory case series, we provide a detailed kinematic characterization of the ‘bradykinesia complex’ in three patients with GBA1-associated PD carrying variants of different severity. Unlike idiopathic PD, in which kinematic abnormalities tend to show a relatively consistent pattern with overall predictable dopaminergic responsiveness [4], our findings suggest that GBA1-associated PD may show more heterogeneous motor profiles and variable response to dopaminergic therapy.

The patient carrying the severe L444P variant showed persistent abnormalities despite apparent clinical responsiveness. The apparent dissociation between clinical improvement on the MDS-UPDRS Part III and persistence of abnormalities in selected kinematic parameters after levodopa is consistent with the current conceptualization of bradykinesia as a multidimensional motor construct [4]. Although the MDS-UPDRS Part III remains clinically valuable, it may not capture subtle motor features, whereas kinematic analysis provides a more objective and fine-grained assessment of movement [4]. Accordingly, dopaminergic therapy may improve overall motor performance without fully normalizing all kinematic abnormalities.

Furthermore, while severe GBA1 variants are generally associated with greater cognitive and neuropsychiatric burden, the patient carrying the L444P variant did not show prominent non-motor impairment at the time of evaluation. This finding should be interpreted cautiously, as it may reflect phenotypic variability or a stage preceding overt non-motor clinical expression, thus requiring longitudinal observation [1], [2], [5], [6].

By contrast, the patient carrying the mild N409S variant exhibited widespread kinematic impairment in the OFF state, accompanied by marked improvement after dopaminergic treatment. This pattern is in line with previous evidence suggesting that mild GBA1 variants may be associated with relatively preserved dopaminergic responsiveness and a more favorable motor trajectory [1], [2].

The patient carrying the intermediate-severity R296Q variant showed limited dopaminergic responsiveness across several kinematic domains, despite short disease duration and preserved global cognition. Notably, the presence of affective symptoms may suggest early involvement of limbic and associative circuits, consistent with disruption of motor control mechanisms driven by widespread network impairment [5], [6].

Taken together, these cases illustrate heterogeneity in kinematic motor features and dopaminergic responsiveness in GBA1-associated PD, and suggest that genotype may be one of several factors contributing to this variability. Systematic reviews and large genetic databases have highlighted substantial genotype–phenotype heterogeneity in GBA-PD, with more severe variants being associated with greater motor and non-motor burden [1], [2], [5], [6]. Our findings extend these observations by suggesting that variability in movement patterns may represent an additional dimension of heterogeneity in GBA1-associated PD.

Our findings are also consistent with data from large longitudinal and multimodal cohorts, which indicate that biological and imaging markers may reveal alterations before overt clinical divergence [6]. Quantitative motor analysis may represent a complementary tool within this multidimensional framework. It may help identify patients more likely to benefit from dopaminergic therapy and support stratification in future disease-modifying trials targeting glucocerebrosidase-related pathways. It may also contribute to the longitudinal monitoring of patients who could be candidates for advanced therapeutic strategies, such as deep brain stimulation [7].

Although genotype may have contributed to the observed differences, the variability across cases likely reflects the interplay of multiple factors, including disease duration, medication exposure, cognitive and psychiatric features, and imaging findings, all of which may have influenced kinematic performance and levodopa responsiveness. Nevertheless, these preliminary observations may support future studies exploring whether distinct GBA1 variants are associated with different kinematic patterns and treatment-response profiles. Confirmation in adequately powered longitudinal studies will be needed to more robustly define genotype–phenotype relationships and their prognostic relevance. In conclusion, the kinematic assessment of these three cases suggests that motor impairment and dopaminergic responsiveness in GBA1-associated PD may be heterogeneous. Quantitative kinematic analysis may provide an additional layer of phenotypic characterization, warranting further evaluation in larger longitudinal studies.

## CRediT authorship contribution statement

**Martina De Riggi:** Writing – review & editing, Writing – original draft, Visualization, Validation, Software, Resources, Project administration, Methodology, Investigation, Formal analysis, Data curation, Conceptualization. **Daniele Birreci:** Writing – review & editing, Methodology, Investigation. **Adriana Martini:** Writing – review & editing. **Simone Aloisio:** Writing – review & editing. **Luca Angelini:** Writing – review & editing. **Anna Sofia Grandolfo:** Writing – review & editing. **Matteo Bologna:** Writing – review & editing, Supervision, Conceptualization.

## Declaration of competing interest

The authors declare that they have no known competing financial interests or personal relationships that could have appeared to influence the work reported in this paper.
