# Supplementary material for: Analyzing the ‘bradykinesia complex’ in GBA1-associated Parkinson's disease: A series of three cases
Source: Clin Park Relat Disord. 2026 Jun 11;15:100462. doi: 10.1016/j.prdoa.2026.100462 (PMC13292783; doi:10.1016/j.prdoa.2026.100462)
Supplement: Supplementary file 1 — Supplementary material [file mmc1.docx]

**MATERIALS AND METHODS**

**Participants**

Three patients with PD were recruited for this study at the Department of Human Neurosciences, Sapienza, University of Rome, Italy. Patients were evaluated with the Movement Disorder Society–sponsored revision of the Unified Parkinson’s Disease Rating Scale (MDS-UPDRS) [1] part III, performed with a standardized examination protocol. Given the asymmetric motor involvement typical of PD, the most affected side was selected for kinematic analysis [12]. To define the most affected side in patients, we considered the sum of items 3.3–3.8 for each side [2]. These specific items regard the evaluation of rigidity and repetitive movements of the upper and lower limbs [1]. All participants underwent a cognitive evaluation using the Montreal Cognitive Assessment (MoCA) [3] and the Frontal Assessment Battery (FAB) [4]. Clinical assessment in PD also included the Hoehn and Yahr scale [5], the Hamilton depression rating scale (HAM-D) [6], the Hamilton anxiety rating scale (HAM-A) [7], and the Fatigue Severity Scale (FSS) [8]. Levodopa equivalent daily dose (LEDD) was calculated in patients [9].

**Kinematic recording and analysis**

Patients participated in two experimental sessions: i) under their usual therapeutic regimen (ON condition), ii) after overnight withdrawal (at least 12 h) of their medications (OFF condition) [10,11]. We recorded the kinematics of finger tapping using a 3D optoelectronic system (SMART motion system, BTS, Milan, Italy). The system comprises three infrared cameras (sampling rate, 120 Hz) that follow the 3D displacement of reflective markers (5mm in diameter) of negligible weight taped to the participant’s hand, as detailed elsewhere [10,13–16]. Participants were comfortably seated in a chair and were instructed to tap their index finger repetitively on their thumb for 15sec. Three 15sec trials were recorded for each side in a randomized order. Participants were allowed to rest for 45-60sec between acquisition trials to avoid fatigue [10,13–16]. Kinematic data were analyzed using a dedicated software that reconstructs the 3D space displacements of the reflective markers off-line (SMART Analyzer, BTS, Milan, Italy) and determines the kinematic variables of interest, including the number of movements, movement amplitude (degrees) and velocity (degrees/s), and movement rhythm, represented by the coefficient of variation (CV) computed by the standard deviation/mean value of the inter-tap intervals (with higher values representing lower regularity of repetitive movements) [10,13–16]. Linear regression techniques were used to determine the intercept (reflecting movement amplitude in degrees and velocity in degrees/s at the beginning of the 15-s motor sequence) and slope (representing amplitude and velocity decrement, i.e., sequence effect across the 15-s trials) of the regression line across the scatter plot of the kinematic parameters (y-axis) versus the number of movements (x-axis) [10,13–16].

**Genetic analysis**

Genetic testing was performed on genomic DNA extracted from peripheral blood. Next-generation sequencing (NGS) was used to analyze a panel of genes associated with early-onset Parkinson’s disease, including ATP13A2, CHCHD2, DNAJC6, EIF4G1, FBXO7, GBA, LRRK2, MAPT, PARK7, PINK1, PLA2G6, POLG, PRKN, RAB39B, SNCA, SYNJ1, SLC6A3, VPS13C, and VPS35. The analytical sensitivity of the assay was approximately 95%.

In addition, targeted analysis was performed for exon deletions and/or multiplications involving SNCA (exons 2a, 4–6), PRKN/Parkin (exons 1–12), PINK1 (exons 1–8), PARK7/DJ-1 (exons 1b, 3, 5, 7), and ATP13A2 (exons 2 and 9). Targeted testing was also carried out for the recurrent mutations LRRK2 p.G2019S and SNCA p.A30P.

**SUPPLEMENTARY TABLES**

|  | **Case 1** | **Case 2** | **Case 3** |
| --- | --- | --- | --- |
| Visuospatial/Executive | 5(5) | 4(4) | 5(5) |
| Naming | 3(3) | 3(3) | 3(3) |
| Attention | 6(6) | 5(5) | 6(6) |
| Language | 3(3) | 3(3) | 3(3) |
| Abstraction | 2(2) | 2(2) | 2(2) |
| Delayed Recall | 4(4) | 1(1) | 5(5) |
| Orientation | 6(6) | 6(6) | 6(6) |
| **Total Score** | **29(29)** | **24(24)** | **30(30)** |

**Supplementary Table 1.** Montreal Cognitive Assessment (MoCA) total scores and domain-specific subscores in the three patients with GBA1-associated Parkinson’s disease (PD). Scores are presented as OFF medication (outside parentheses) and ON medication (within parentheses).

**BIBLIOGRAPHY**

[1] C.G. Goetz, B.C. Tilley, S.R. Shaftman, G.T. Stebbins, S. Fahn, P. Martinez‐Martin, W. Poewe, C. Sampaio, M.B. Stern, R. Dodel, B. Dubois, R. Holloway, J. Jankovic, J. Kulisevsky, A.E. Lang, A. Lees, S. Leurgans, P.A. LeWitt, D. Nyenhuis, C.W. Olanow, O. Rascol, A. Schrag, J.A. Teresi, J.J. Van Hilten, N. LaPelle, Movement Disorder Society‐sponsored revision of the Unified Parkinson’s Disease Rating Scale (MDS‐UPDRS): Scale presentation and clinimetric testing results, Mov. Disord. 23 (2008) 2129–2170. https://doi.org/10.1002/mds.22340.

[2] M. Kojovic, M. Bologna, P. Kassavetis, N. Murase, F.J. Palomar, A. Berardelli, J.C. Rothwell, M.J. Edwards, K.P. Bhatia, Functional reorganization of sensorimotor cortex in early Parkinson disease, Neurology 78 (2012) 1441–1448. https://doi.org/10.1212/WNL.0b013e318253d5dd.

[3] Z.S. Nasreddine, N.A. Phillips, V. Bédirian, S. Charbonneau, V. Whitehead, I. Collin, J.L. Cummings, H. Chertkow, The Montreal Cognitive Assessment, MoCA: a brief screening tool for mild cognitive impairment, J. Am. Geriatr. Soc. 53 (2005) 695–699. https://doi.org/10.1111/j.1532-5415.2005.53221.x.

[4] B. Dubois, A. Slachevsky, I. Litvan, B. Pillon, The FAB: a Frontal Assessment Battery at bedside, Neurology 55 (2000) 1621–1626.

[5] M.M. Hoehn, M.D. Yahr, Parkinsonism: onset, progression, and mortality, Neurology 17 (1967) 427–427. https://doi.org/10.1212/WNL.17.5.427.

[6] M. Hamilton, A rating scale for depression, J. Neurol. Neurosurg. Psychiatry 23 (1960) 56–62.

[7] M. Hamilton, The assessment of anxiety states by rating, Br. J. Med. Psychol. 32 (1959) 50–55.

[8] J.H. Friedman, G. Alves, P. Hagell, J. Marinus, L. Marsh, P. Martinez-Martin, C.G. Goetz, W. Poewe, O. Rascol, C. Sampaio, G. Stebbins, A. Schrag, Fatigue rating scales critique and recommendations by the Movement Disorders Society task force on rating scales for Parkinson’s disease, Mov. Disord. Off. J. Mov. Disord. Soc. 25 (2010) 805–822. https://doi.org/10.1002/mds.22989.

[9] S.T. Jost, M.-A. Kaldenbach, A. Antonini, P. Martinez-Martin, L. Timmermann, P. Odin, R. Katzenschlager, R. Borgohain, A. Fasano, F. Stocchi, N. Hattori, P.L. Kukkle, M. Rodríguez-Violante, C. Falup-Pecurariu, S. Schade, J.N. Petry-Schmelzer, V. Metta, D. Weintraub, G. Deuschl, A.J. Espay, E.-K. Tan, R. Bhidayasiri, V.S.C. Fung, F. Cardoso, C. Trenkwalder, P. Jenner, K. Ray Chaudhuri, H.S. Dafsari, International Parkinson and Movement Disorders Society Non-Motor Parkinson Disease Study Group, Levodopa Dose Equivalency in Parkinson’s Disease: Updated Systematic Review and Proposals, Mov. Disord. Off. J. Mov. Disord. Soc. 38 (2023) 1236–1252. https://doi.org/10.1002/mds.29410.

[10] M. Bologna, A. Guerra, G. Paparella, L. Giordo, D. Alunni Fegatelli, A.R. Vestri, J.C. Rothwell, A. Berardelli, Neurophysiological correlates of bradykinesia in Parkinson’s disease, Brain J. Neurol. (2018). https://doi.org/10.1093/brain/awy155.

[11] A. Guerra, D. Colella, M. Giangrosso, A. Cannavacciuolo, G. Paparella, G. Fabbrini, A. Suppa, A. Berardelli, M. Bologna, Driving motor cortex oscillations modulates bradykinesia in Parkinson’s disease, Brain J. Neurol. 145 (2022) 224–236. https://doi.org/10.1093/brain/awab257.

[12] Z. Wu, H. Gu, R. Hong, Z. Xing, Z. Zhang, K. Peng, Y. He, L. Xie, J. Zhang, Y. Gao, Y. Jin, X. Su, H. Zhi, Q. Guan, L. Pan, L. Jin, Kinect-based objective evaluation of bradykinesia in patients with Parkinson’s disease, Digit. Health 9 (2023) 20552076231176653. https://doi.org/10.1177/20552076231176653.

[13] D. Colella, A. Guerra, G. Paparella, E. Cioffi, A. Di Vita, A. Trebbastoni, A. Berardelli, M. Bologna, Motor dysfunction in mild cognitive impairment as tested by kinematic analysis and transcranial magnetic stimulation, Clin. Neurophysiol. Off. J. Int. Fed. Clin. Neurophysiol. 132 (2021) 315–322. https://doi.org/10.1016/j.clinph.2020.10.028.

[14] A. Guerra, D. Colella, M. Giangrosso, A. Cannavacciuolo, G. Paparella, G. Fabbrini, A. Suppa, A. Berardelli, M. Bologna, Driving motor cortex oscillations modulates bradykinesia in Parkinson’s disease, Brain (2021) awab257. https://doi.org/10.1093/brain/awab257.

[15] G. Paparella, M. Ceccanti, D. Colella, A. Cannavacciuolo, A. Guerra, M. Inghilleri, A. Berardelli, M. Bologna, Bradykinesia in motoneuron diseases, Clin. Neurophysiol. Off. J. Int. Fed. Clin. Neurophysiol. 132 (2021) 2558–2566. https://doi.org/10.1016/j.clinph.2021.08.006.

[16] A. De Biase, G. Paparella, L. Angelini, A. Cannavacciuolo, D. Colella, E. Cerulli Irelli, A.T. Giallonardo, C. Di Bonaventura, A. Berardelli, M. Bologna, Tremor and Movement Slowness Are Two Unrelated Adverse Effects Induced by Valproate Intake, Mov. Disord. Clin. Pract. 9 (2022) 1062–1073. https://doi.org/10.1002/mdc3.13560.
